# Supplementary material for: The interactome of intact mitochondria by cross-linking mass spectrometry provides evidence for coexisting respiratory supercomplexes
Source: Mol Cell Proteomics. 2017 Dec 8;17(2):216–32. doi: 10.1074/mcp.RA117.000470 (PMC5795388; doi:10.1074/mcp.RA117.000470)
Supplement: Supplemental Data [file supp_RA117.000470_133922_0_supp_23978_fzffwf.zip › spectra_annotation/mito_DR_spectra_annotation/258-1-15-1-20-1.pdf]

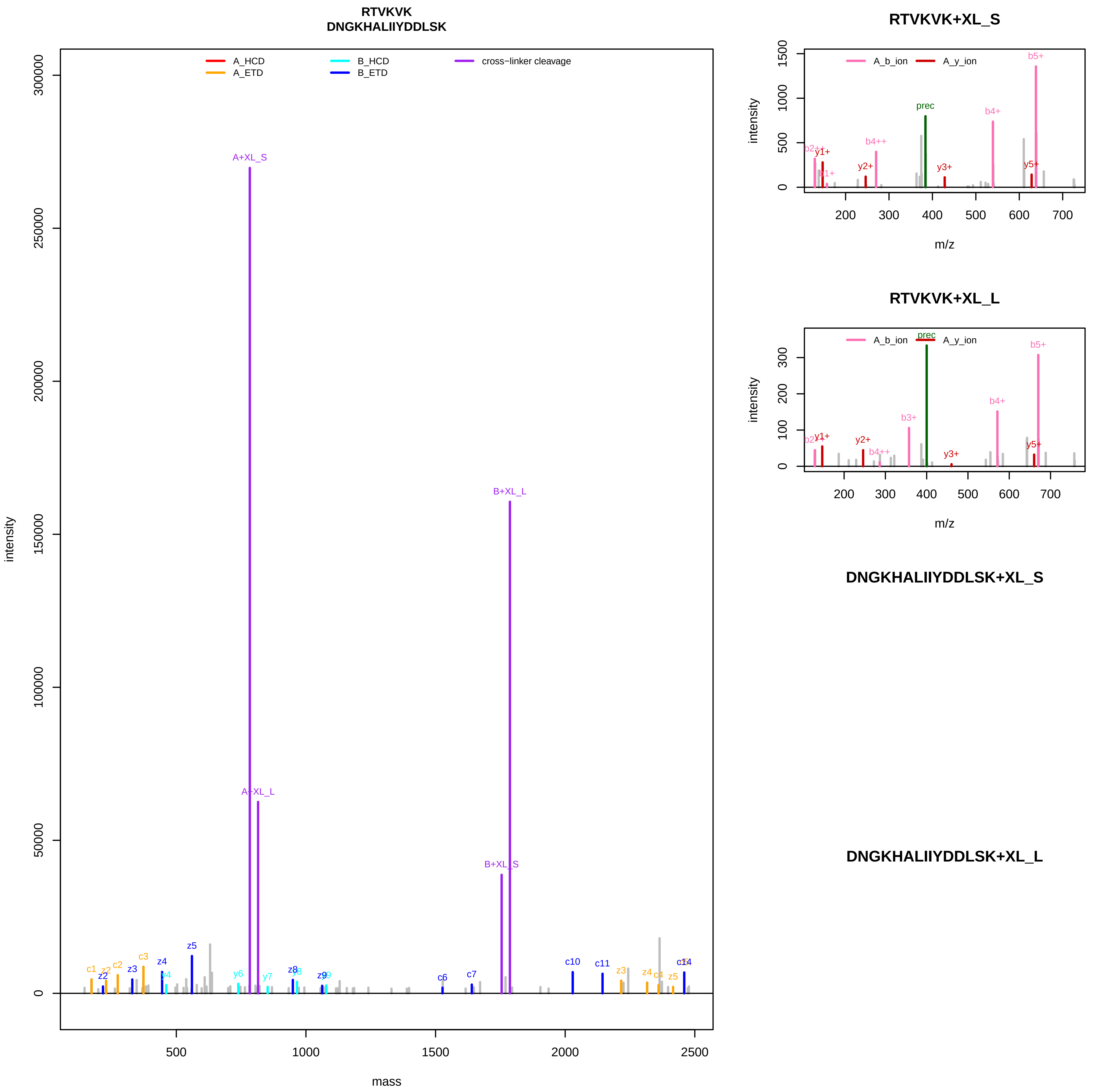
RTVKVK
DNGKHALIIYDDLSK
A_HCD
A_ETD
B_HCD
B_ETD
cross−linker cleavage
A+XL_S
A+XL_L
B+XL_L
B+XL_S
intensity
mass
0
50000
100000
150000
200000
250000
300000
500
1000
1500
2000
2500
c1
z2
c2
z3
c3
z4
y4
z5
y6
y7
z8
z9
c6
c7
c10
c11
z3
z4
c4
z5
c14
RTVKVK+XL_S
A_b_ion
A_y_ion
prec
b5+
b4+
b4++
b2++
y1+
b1+
y2+
y3+
y5+
intensity
m/z
RTVKVK+XL_L
A_b_ion
A_y_ion
prec
b5+
b4+
b3+
b4++
b2++
y1+
y2+
y3+
y5+
intensity
m/z
DNGKHALIIYDDLSK+XL_S
DNGKHALIIYDDLSK+XL_L
